# The Role of Transglutaminase 2 in the Radioresistance of Melanoma Cells

**DOI:** 10.3390/cells11081342

**Published:** 2022-04-14

**Authors:** Julia Aepler, Johanna Wodtke, Robert Wodtke, Cathleen Haase-Kohn, Reik Löser, Jens Pietzsch, Sandra Hauser

**Affiliations:** 1 Helmholtz-Zentrum Dresden-Rossendorf, Institute of Radiopharmaceutical Cancer Research, Bautzner Landstrasse 400, 01328 Dresden, Germany; j.aepler@hzdr.de (J.A.); j.wodtke@hzdr.de (J.W.); r.wodtke@hzdr.de (R.W.); c.haase@hzdr.de (C.H.-K.); r.loeser@hzdr.de (R.L.); j.pietzsch@hzdr.de (J.P.); 2 School of Sciences, Faculty of Chemistry and Food Chemistry, Technische Universität Dresden, Mommsenstrasse 4, 01307 Dresden, Germany

**Keywords:** clonal expansion, fluorescence anisotropy assay, malignant melanoma, tumor radioresistance, TG2 inhibition

## Abstract

Transglutaminase 2 (TG2) is a protein expressed in many tissues that exerts numerous, sometimes contradictory, intra- and extracellular functions, under both physiological and pathophysiological conditions. In the context of tumor progression, it has been found to be involved in cell adhesion, DNA repair mechanisms, induction of apoptosis, and mesenchymal transdifferentiation, among others. Here, we hypothesized that TG2 also contributes to the radioresistance of two human melanoma cell lines, A375 and MeWo, which can be seen to differ in their basal TG2 biosynthesis by examining their proliferation and clonal expansion after irradiation. For this purpose, cellular TG2 biosynthesis and TG2 activity were modulated by transfection-induced overexpression or TG2 knock-out and application of TG2-selective inhibitors. Proliferation and clonal expansion of TG2-overexpressing cells was not enhanced over wildtype cells, suggesting that increased TG2 biosynthesis does not further enhance the radioresistance of melanoma cells. Conversely, TG2 knock-out in A375 cells reduced their proliferation, as well as clonal and spheroidal expansion after irradiation, which indicates a contribution of TG2 to the radioresistance of melanoma cells. Since TG1, TG3, and partly also, TG6 biosynthesis was detectable in A375 and MeWo cells, it can be assumed that these other members of the TG family may exert a partially compensatory effect.

## 1. Introduction

Transglutaminase (TG) 2, an acyltransferase (EC 2.3.2.13), which is also referred to as tissue transglutaminase, catalyzes the transamidation between γ-carboxy amide groups of protein-bound glutamine-residues (acyl donor) and a variety of primary amines (acyl acceptor). For example, the intra- or intermolecular crosslinking of proteins results from the assembly of *N*^ε^-(γ-glutamyl-) lysine bonds. Besides the catalytic core that also covers the calcium binding sites, the protein structure contains binding motifs for fibronectin, GTP/GDP, and phospholipase C and exhibits other enzyme functions, such as GTP/ATPase, protein kinase, disulfide isomerase, and DNA nuclease [1]. GTP and calcium ions act as allosteric mediators of the transamidase activity (TAA) [2,3,4]. The binding of GTP inhibits the TAA via stabilization of the closed protein conformation, whereas the binding of calcium ions promotes the open and catalytically active protein conformation [5,6]. Consequently, under physiological conditions, TG2 remains predominantly inactive due to the high intracellular GTP availability, but enables a rapid response in the event of disturbances in cellular calcium homeostasis [1]. For example, TG2 TAA activation as a result of stress-related calcium ion influx in the cell leads to cross-linkage of transcription factor SP1 and thereby, to induction of apoptosis [7,8]. Because of the complex TG2 promotor structure, the TG2 transcription is subject to a multi-factor regulation, including retinoic acid, IL-6, TNF-α, and TGF-β1 [9,10,11,12]. In addition, NF-κB regulates TG2 transcription as a reaction to genotoxic stress [13]. Moreover, this process was found to be self-enhanced by TG2-mediated polymerization and, thus, inactivation of I-κBα, the inhibitory α-subunit of NF-κB [14].

Even though TG2 is mainly located in the cytoplasm, it can be translocated into the nucleus via an importin-α3-related signaling pathway, where it facilitates the transcription of cell survival genes [15] and DNA damage repair via interaction with topoisomerase IIα [16]. Furthermore, TG2 is secreted into the extracellular space by an unconventional mechanism mediated by P2X7 receptor activation, as well as endosomal and plasma membrane-originated vesicles [17,18]. Once in the extracellular space, TG2 is involved in multiple extracellular matrix (ECM)-related processes via the TAA, as well as via non-enzymatic functions, including the enhancement of the ECM stability by cross-linkage of ECM components, such as fibronectin or collagen [19,20,21,22], and the organization of stable junctions between cells and ECM components. In particular, TG2 functions as a co-receptor for integrin and fibronectin, which facilitates cell adhesion and cellular survival (via activation of FAK) [23,24,25]. Furthermore, the association of integrins and the PDGF receptor is enhanced by TG2 action, which leads to increased activation of AKT-1 and SHP-2 in the presence of PDGF and thereby, facilitates cell proliferation and migration [26]. In addition, the involvement of TG2 in several other pro-tumorigenic signal transduction pathways was identified for certain cancer cell lines, although its particular function at the molecular level often remains elusive [27].

Interestingly, TG2 biosynthesis was found, to a larger extent, in metastasizing and chemoresistant tumors than in primary tumors of various cancer types, suggesting that TG2 is involved in the progression, rather than in the development of tumors [28,29]. With regard to tumor progression, the mesenchymal transdifferentiation (MT) plays an important role, and a contribution of TG2 to this process via regulation of C/EBPb signaling was demonstrated by Yin et al. in 2017 [30], and is also being discussed because of the TG2 activation potential for TGF-β1, FAK and AKT, which represent well-known mediators of the MT [2,28]. In correlation with its involvement in MT, but also cell survival and DNA repair mechanisms, TG2 was found to affect the therapy-resistant phenotype of different cancer types [29,31,32], including malignant melanoma, an aggressive tumor, highly resistant to standard chemo- and radiotherapy. Because of the lack of successful treatment options for melanoma, there is an emerging need for the identification of cellular structures that can be targeted in order to sensitize tumor cells towards anticancer therapy [33].

In patients suffering from laryngeal cancer, low expression of TG2 (combined with high expression of BNIP3) was identified as a prognostic factor for survival after postoperative radiotherapy [34]. Furthermore, TG2-deficient mice showed reduced UV-irradiation-induced inflammation [35] and TG2 derived from irradiated astrocytes promoted the stemness of glioma cells [36]. Taking these findings into account, a contribution of this enzyme to the radioresistance of tumor cells is hypothesized.

The attenuated sensitivity of melanoma cells to ionizing radiation has been reported within several studies [37,38]. Similar to the cellular effects of ionizing radiation, alkylating cytostatic agents act by compromising the structural integrity of DNA [39]. In this context, the increased expression and activity of TG2 in melanoma has been linked to impaired sensitivity towards the electrophilic cytostatic agents, cisplatin and dacarbazine [29]. Besides this tumor progression function, TG2 was shown to suppress melanoma metastasis by the polyamination of matrix proteins [40]. Other studies defined tumor-suppressive functions of TG2 in melanoma by identifying it as a ligand for the adhesion G protein-coupled receptor GRP56 [40], which leads to receptor-mediated internalization and subsequent intracellular degradation of the enzyme [41].

Motivated by the increased radioresistance of melanoma and the mentioned controversial results regarding the function of TG2 in this tumor entity, we investigated the contribution of TG2 to the radioresistance of melanoma cells. For this purpose, we examined the proliferation and clonal expansion of irradiated melanoma cells with different TG2 expression levels (TG2-positive: A375, TG2-negative: MeWo) in 2D and 3D cultures. Furthermore, cellular TG2 biosynthesis was modified by transfection-induced overexpression of TG2, as well as TG2 knock-out (KO), and TG2 activity was modulated by the application of TG2-selective inhibitors.

## 2. Materials and Methods

### 2.1. Generation and Cultivation of Melanoma Sub-Cell Lines

Human melanoma cell lines A375 (# CRL-1619) and MeWo (# HTB-65) were obtained from the American Tissue Culture Collection (Manassas, VA, USA) and were modified via the lentiviral pHATrick vector to express fluorophore mCherry (“-control”) and subsequently with human *TGM2* gene to also express human TG2 (“-TG2”) following a protocol published by Müller et al. in 2015 [42].

Furthermore, in A375 cells, TG2 was knocked out using CRISPR/Cas9 technology. For this aim, CRISPR/Cas9 knockout plasmids against human TG2 were purchased from Santa Cruz Biotechnology (Heidelberg, Germany). Transfection was performed as recommended by the manufacturer. Briefly, 2 × 10^5^ A375 cells were seeded 24 h prior to transfection. UltraCruz^®^ Transfection Reagent (sc-395739, Santa Cruz Biotechnology, Heidelberg, Germany) was used at a final concentration of 1% together with a total of 4 µg plasmid DNA (2 µg *TGase2 CRISPR/Cas9 KO* plasmid (sc-401543) with 2 µg *TGase2 HDR* plasmid (sc-401543-HDR)) per well (all plasmids from Santa Cruz Biotechnology, Heidelberg, Germany). The plasmid DNA/UltraCruz^®^ complexes were made up in serum-free growth medium. Cells were maintained for 24 h before returning to growth medium and selection with 0.5 µg/mL puromycin (Santa Cruz Biotechnology, Heidelberg, Germany). During the repair of the site-specific Cas9-induced DNA cleavage within the *TGM2* gene, the *TGase2 HDR* plasmid incorporates a puromycin resistance gene to enable selection of stable KO cells and an *RFP* gene to visually confirm transfection. RFP positive and puromycin resistant cells were selected by collection of monoclonal populations (“-KO TG2”). Successful KO was verified by Western blot analysis.

Tumor cells were maintained in monolayer cultures under standard conditions using Dulbecco’s Modified Eagle’s Medium supplemented with 10% (*v*/*v*) fetal bovine serum and 100 U/mL penicillin/streptomycin (all reagents from Biochrom, Berlin, Germany). Contaminations with mycoplasma were excluded on a regular basis by application of Venor^®^GeM Classic kit (Minerva Biolabs, Berlin, Germany) according to manufacturer’s instructions.

### 2.2. Treatment of Melanoma Sub-Cell Lines

Two irreversible inhibitors, *N*^α^-Phenylacetyl-*N*^ε^-acryloyl-l-lysine-4-(6-nitropyridin-2-yl) piperazide (**I1**) and *N*^α^-Phenylacetyl-*N*^ε^-acryloyl-l-lysine-4-(pyridin-4-yl) piperazide (**I2**), described by Wodtke et al. in 2018 [43], were used to modify TG2 activity (structures shown in Figure 4). TG2 inhibitors were applied as a single dose of 10 µM or the same final concentration of the solvent DMSO (Sigma-Aldrich, Steinheim, Germany), 0.25% (*v*/*v*) or 35.2 mM, was applied as a control. The treatment persisted until the termination of the experiment without medium exchange or further TG2 inhibitor administration. CellTiter-Blue^®^ (Promega, Mannheim, Germany) and Pierce™ LDH assay (Thermo Fisher Scientific, Rockford, IL, USA) were applied to determine cell viability and cytotoxicity of inhibitors to human melanoma cell lines, respectively, according to manufacturer’s instructions. Triton^®^-X-100 (2% (*v*/*v*), Sigma-Aldrich, Steinheim, Germany) served as a cytotoxic reference.

Cell irradiation, with or without prior TG2 inhibitor treatment, was performed at room temperature with single doses of 200 kV X-rays filtered with 0.5 mm copper (Maxishot, Yxlon International, Hamburg, Germany). The absorbed dose was measured using a UNIDOS dosimeter (PTW, Freiburg, Germany) and dose rate was approximately 1.1 Gy/min at 20 mA. Non-irradiated control groups were simultaneously kept at room temperature to obtain comparable conditions.

### 2.3. Proliferation and Clonal Expansion

For 2D cultures, 24 h prior to treatment with 10 µM of TG2 inhibitors or with DMSO and irradiation, all melanoma cell lines were sub-cultivated at specified cell numbers dependent on assay type, cell line and intended treatment (Table 1). For proliferation assessment, living cells were detached via trypsin/EDTA (Biochrom, Berlin, Germany) and counted using CASY cell-counting device (Roche Innovatis GmbH, Mannheim, Germany) 24, 48 and 72 h after treatment. To determine survival fractions, cell counts were normalized to untreated controls. Doubling times were calculated via exponential regression using Prism version 9.1.2 (GraphPad Software, San Diego, CA, USA). Clonal expansion was examined by microscopy-assisted counting of cell colonies consisting of more than 50 cells, fixated with methanol and stained with crystal violet after 120 h (A375) or 336 h (MeWo) of cultivation. Plating efficiencies were determined as colony counts per inserted cell number in order to be used to calculate survival fractions, such as for proliferation assessment.

### 2.4. Spheroidal Expansion

For multicellular 3D cultures, 1 × 10^3^ A375-WT and -KO TG2 cells were sub-cultivated in plates with ultra-low-attachment surface (PerkinElmer, Waltham, MA, USA). After 72 h, cells were treated with 10 µM of TG2 inhibitors or with DMSO and irradiated as described above. The spheroidal expansion was observed routinely for 14 days by automated brightfield and phase contrast microscopy using the live-cell analysis system IncuCyte (Sartorius AG, Göttingen, Germany). The spheroidal area was determined with Rover version 3.0.60h (ABX GmbH, Radeberg, Germany) in regions of interest drawn via semi-automated contrast-based detection applying a threshold of between 0 and 180. Identified regions of interest were checked for correctness and adjusted manually if necessary.

### 2.5. Western Blot Analysis

At the same time when cellular material was collected as described for the proliferation assessment, cell culture supernatants were sampled for Western blot analysis. Cell lysates and cell-free supernatants were obtained as described previously [44]. Protein concentrations of cell lysates were determined in order to separate equal protein amounts by SDS-PAGE and immobilize to PVDF membranes by electroblotting as described elsewhere [45]. Biosynthesis of TGs and ß-actin as well as secretion of TG2 were analyzed via incubation of Western blots with target-specific primary antibodies, peroxidase-coupled secondary antibodies (Table 2) and SuperSignal™ West Chemiluminescent substrates (Thermo Fisher Scientific, Rockford, IL, USA). The densitometric indices were calculated for each sample as the quotient of the band intensity of the protein of interest and the loading control ß-actin using the software TotalLab TL100 1D version 2008.01 (Nonlinear Dynamics, Newcastle upon Tyne, UK). Subsequently, densitometric indices of treated samples were normalized to untreated controls.

### 2.6. In Vitro TG2 Activity

**Figure 1:**
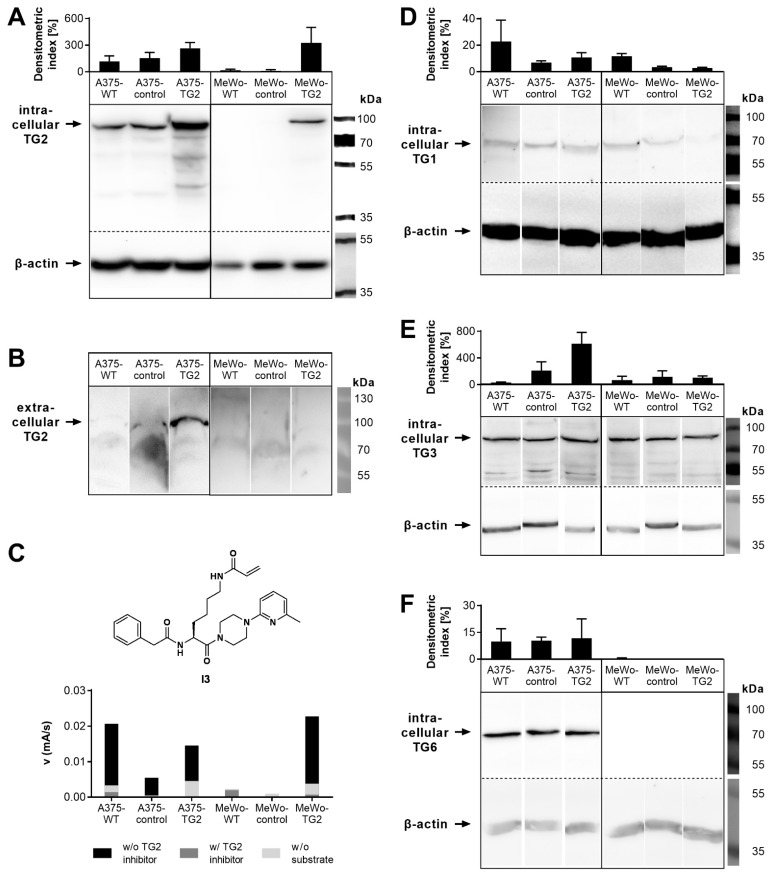
Biosynthesis of transglutaminases (TG) as well as secretion and cellular in vitro activity of TG2 in melanoma sub-cell lines. (**A**,**B**) TG2 biosynthesis (**A**) and secretion (**B**) were determined via Western blot using cell lysates and cell culture supernatants, respectively, and TG2 biosynthesis was analyzed using semi-quantitative densitometry. Densitometric indices were gained by normalization to respective ß-actin biosynthesis values. (**C**) Overall TG activity (black bars), remaining activity after selective inhibition using TG2 inhibitor **I3** (dark grey bars, structure is shown above [47]), and baseline activity without substrate (light grey bars) as measured in vitro by fluorescence anisotropy assay in cell lysates. Data for A375-WT and MeWo-WT were published previously [43]. (**D**–**F**) Biosynthesis of TG1 (**D**), TG3 (**E**) and TG6 (**F**) was determined via Western blot and semi-quantitative densitometric analysis. Densitometric indices were gained by normalization to respective ß-actin biosynthesis values. Representative blots as well as mean values ± SD from individual experiments (*n* ≥ 3, except from in vitro activity measurements (*n* = 2, no SD applicable)) are shown.

In vitro TG2 activity was determined by fluorescence anisotropy (FA) assay and performed as described previously [43,46]. Measurements of TG2 activity in cell lysates (preparation as described for Western blot analysis using modified lysis buffer, containing 150 mM NaCl; 50 mM Tris, pH 8.0; 1 μg/mL Leupeptin; 1 mM PMSF; 5 mM NaF; 1 mM Na_3_VO_4_ and 1 mM DTT) were conducted at 30 °C over 900 s with intervals of 30 s using Synergy 4 Multi-Mode Microplate Reader and Gen 5 software (BioTek Instruments, Winooski, VT, USA) and black 96-well F-bottom plates (BRAND, Wertheim, Germany) at an excitation wavelength of 540 nm and emission wavelength of 620 nm. 20 µL of assay buffer, containing 100 mM MOPS, 6 mM CaCl_2_ and 50 μM EDTA and adjusted to pH 8.0 with 1 M NaOH, and 2.5 µL of DMSO or TG2 inhibitor *N*^α^-Phenylacetyl-*N*^ε^-acryloyl-l-lysine-4-(6-methylpyridin-2-yl) piperazide (**I3**, 10 µM in DMSO, structure shown in Figure 1C [47]) was added to 50 µL of cell lysate (2 g protein/L), and the mixture was incubated for 10 min at 30 °C. The reaction was initiated upon addition of 2.5 µL of R-I-cadaverine (0.81 μM in DMSO) as acyl acceptor and 25 µL of *N*,*N*-dimethylcasein (DMC, 30 μM in assay buffer) as acyl donor. For control measurements DMC was omitted. Using Prism version 5.04, the recorded time courses were analyzed by linear regression over 900 s, from which the rates of the enzymatic reactions were obtained as slopes of the regression lines.

### 2.7. Statistical Analysis

Data were expressed as mean or mean ± standard deviation (SD), when applicable. Prism version 9.1.2 was utilized to perform one- or two-way ANOVA and Bonferroni *post hoc* tests. *p* values smaller than 0.05 were identified as statistically significant (* *p* ≤ 0.05, ** *p* ≤ 0.01, *** *p* ≤ 0.001, **** *p* ≤ 0.0001). All experiments were performed at least 2 independent times.

## 3. Results

### 3.1. TG Biosynthesis, Secretion and Activity of Melanoma Cells

Lentiviral modification of TG2-expressing A375 cells with the human *TGM2* gene resulted in elevated biosynthesis (overexpression) of cellular TG2, compared to wildtype (A375-WT) cells and transduction control cells (A375-control, Figure 1A). Likewise, artificial TG2 biosynthesis was generated in MeWo cells (MeWo-TG2). Western blot analysis revealed comparable TG2 biosynthesis of A375- and MeWo-TG2 cells. TG2 secreted into the extracellular space was found only in naturally TG2-expressing sub-cell lines (A375, Figure 1B). Because of the unavailability of an extracellular loading control, a quantification of the secreted TG2 was not conducted. Cellular in vitro TG2 activity was detectable in all TG2-positive melanoma sub-cell lines (Figure 1C).

The complementary biosynthesis of TG subtypes TG1, TG3 and TG6 was analyzed (Figure 1D). In A375 sub-cell lines, all tested TG subtypes were detectable, with TG1 and TG6 being present in comparable amounts between sub-cell lines. However, densitometric analysis indicated an intensified TG3 biosynthesis in A375-control cells, as compared to A375-WT cells, and this finding was even more strongly pronounced in A375-TG2 cells. In MeWo sub-cell lines, only biosynthesis of TG1 and TG3 was detectable, and their expression seems to occur to a similar extent within sub-cell lines.

### 3.2. Proliferation, Clonal Expansion and TG2 Biosynthesis of Melanoma Cells after Irradiation

**Figure 2:**
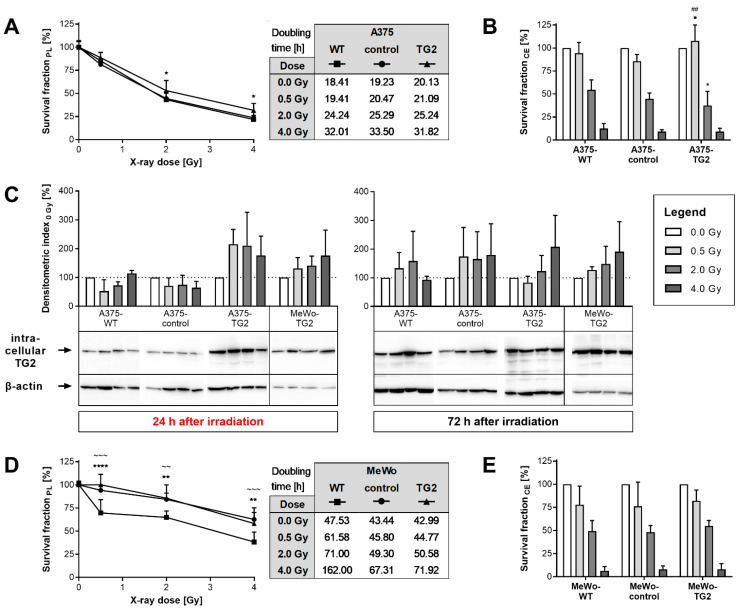
Proliferation, clonal expansion and TG2 biosynthesis of melanoma sub-cell lines after irradiation. (**A**) Proliferation (PL) of A375 sub-cell lines 72 h after irradiation. Survival fractions were calculated from cell counts normalized to non-irradiated (0 Gy) mean values of each cell line. Doubling times (h) over 72 h are indicated in the table. (**B**) Clonal expansion (CE) of A375 sub-cell lines after irradiation. Survival fractions resulted from plating efficiencies normalized to non-irradiated (0 Gy) values. For legend information see quadrangle below. (**C**) TG2 biosynthesis of A375 and MeWo sub-cell lines 24 h (left) and 72 h (right) after irradiation. TG2 biosynthesis was determined via Western blot using cell lysates and semi-quantitative densitometric analysis. Densitometric indices were calculated by normalization to ß-actin biosynthesis values and thereafter to respective non-irradiated (0 Gy) values of each cell line. (**D**) Proliferation (PL) of MeWo sub-cell lines 72 h after irradiation. Survival fractions were calculated from cell counts normalized to non-irradiated (0 Gy) mean values of each cell line. Doubling times (h) over 72 h are indicated in the table. (**E**) Clonal expansion (CE) of MeWo sub-cell lines after irradiation. Survival fractions resulted from plating efficiencies normalized to non-irradiated (0 Gy) values. For legend information see quadrangle above. Mean values ± SD from individual experiments (n ≥ 3) and representative blots are shown. Significant variances are displayed as * (TG2 vs. WT, *p* ≤ 0.05), ** (TG2 vs. WT, *p* ≤ 0.01), **** (TG2 vs. WT, *p* ≤ 0.0001), ^##^ (TG2 vs. control, *p* ≤ 0.01), ^~~^ (control vs. WT, *p* ≤ 0.01), and ^~~~^ (control vs. WT, *p* ≤ 0.001).

The proliferation of A375-TG2 cells, irradiated with 2 or 4 Gy, was increased after 72 h, as compared to A375-WT and -control cells, but not after 24 h (data not shown). However, comparable doubling times were calculated for all A375 sub-cell lines (Figure 2A). The irradiation-dependent clonal expansion of A375-TG2 cells showed controversial results: On the one hand, at a low irradiation dose (0.5 Gy), it was increased, as compared to A375-WT and -control cells. On the other hand, after administration of higher irradiation doses, it was diminished (2 Gy) or similar (4 Gy), as compared to A375-WT cells (Figure 2B). Cellular TG2 biosynthesis of A375-TG2 cells was elevated 24 h after irradiation with 0.5 and 2 Gy, as compared to the non-irradiated control (0 Gy). This effect was absent 72 h after irradiation of A375-TG2 cells and was also not seen in A375-WT and -control cells, at either time point (Figure 2C). Appropriately, the comparison between A375-TG2 cells and A375-WT or -control cells receiving equal irradiation doses indicated an elevation of TG2 biosynthesis at 0.5 and 2 Gy.

Proliferation of MeWo-TG2 and -control cells was elevated compared to MeWo-WT cells 72 h after irradiation, at all applied doses, but not after 24 h (data not shown). Accordingly, irradiation dose-dependent doubling times were reduced (Figure 2D). The irradiation dose-dependent clonal expansion of MeWo-TG2 cells was not affected by artificial TG2 biosynthesis, as compared to MeWo-WT and -control cells (Figure 2E). Further, TG2 biosynthesis in MeWo-TG2 cells was unaffected by irradiation (Figure 2C).

### 3.3. Effects of TG2 Knock-Out on Cellular Reaction towards Irradiation

**Figure 3:**
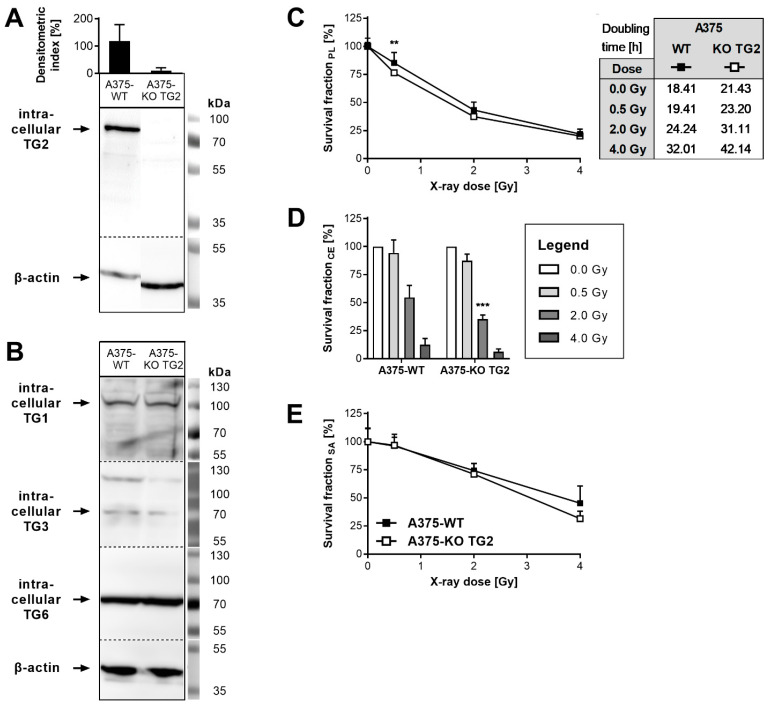
Effects of TG2 knock-out in A375-WT cells on cellular reaction towards irradiation. (**A**) TG2 KO in A375-WT cells was verified via Western blot using cell lysates and semi-quantitative densitometric analysis. Densitometric indices were calculated by normalization to ß-actin biosynthesis values. (**B**) Biosynthesis of TG1, TG3 and TG6 in A375-WT and -KO TG2 cells. ß-actin biosynthesis was employed as a loading control. (**C**) Proliferation (PL) of A375-WT and A375-KO TG2 cells 72 h after irradiation. Survival fractions were calculated from cell counts normalized to non-irradiated (0 Gy) mean values of each cell line. Doubling times (h) over 72 h are indicated in the table. (**D**) Clonal expansion (CE) of A375-WT and A375-KO TG2 cells after irradiation. Survival fractions resulted from plating efficiencies normalized to non-irradiated (0 Gy) values of each cell line. (**E**) Spheroidal areas (SA) were determined at day 14 after irradiation and normalized to respective non-irradiated (0 Gy) mean values. Mean values ± SD (*n* ≥ 4, except of biosynthesis of A375-KO TG2 (*n* = 2)) and representative blots are shown. Significant variances are displayed as ** (KO TG2 vs. WT, *p* ≤ 0.01) and *** (KO TG2 vs. WT, *p* ≤ 0.001).

In addition to those experiments with enhanced cellular TG2 biosynthesis, experiments after the elimination of natural TG2 expression of A375-WT cells by application of CRISPR/Cas9 technology (A375-KO TG2, Figure 3A) were performed. The biosynthesis of other TGs (TG1, TG3 and TG6) was largely unaffected by TG2 KO (Figure 3B), as well as dose-dependent proliferation at 24 h (data not shown). In contrast, proliferation of A375-KO TG2 cells was significantly reduced after 72 h, as compared to A375-WT cells, when cells were irradiated with 0.5 Gy before. Accordingly, basal doubling times of A375-KO TG2 cells were prolonged compared to A375-WT (Figure 3C). Clonal expansion of A375-KO TG2 cells was diminished at all applied irradiation doses, with the most pronounced effect at 2 Gy (Figure 3D). Furthermore, A375-KO TG2 spheroids showed reduced expansion at day 14 after irradiation with 4 Gy, in comparison to A375-WT cells (Figure 3E, not significant).

### 3.4. Effects of TG2 Inhibitors on Cellular Reactions towards Irradiation

**Figure 4:**
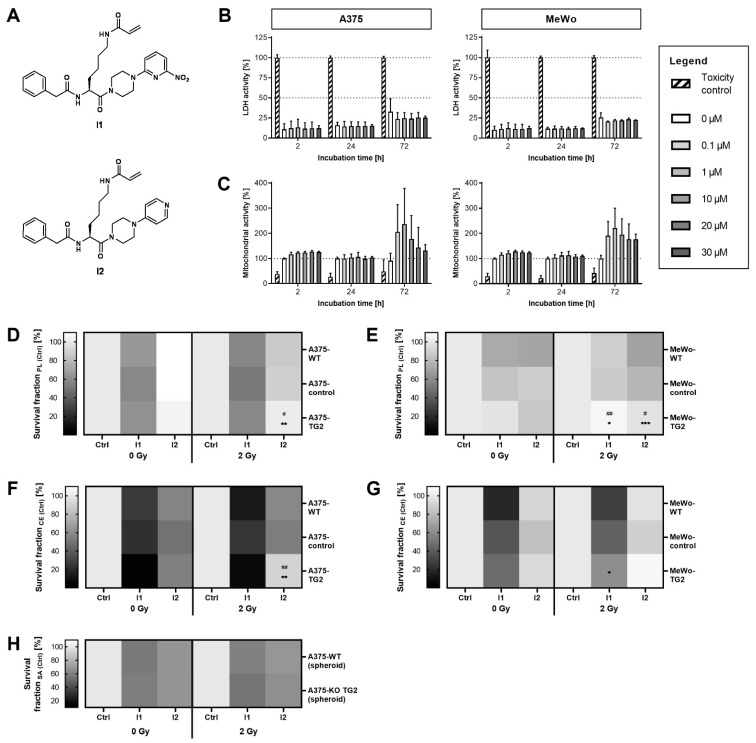
Influence of irreversible TG2 inhibitors on the proliferation and clonal expansion of melanoma sub-cell lines after irradiation. (**A**) Structure of irreversible TG2 inhibitors *N^α^*-Phenylacetyl-*N*^ε^-acryloyl-l-lysine-4-(6-nitropyridin-2-yl) piperazide (**I1**) and *N^α^*-Phenylacetyl-*N*^ε^-acryloyl-l-lysine-4-(pyridin-4-yl) piperazide (**I2**), which exhibit comparable inhibitory potentials but differ in their membrane permeation rates (**I1** >> **I2**) [43]. (**B**,**C**) Toxicity of TG2 inhibitor **I1** on A375- (left) and MeWo-WT (right) cells. Cytotoxicity (**B**) was determined by lactate dehydrogenase (LDH) activity (normalized to toxicity control values) and cell viability (**C**) by mitochondrial activity (normalized to inhibitor-free sample values (0 µM)). (**D**,**E**) Proliferation (PL) after irradiation of A375 (**D**) and MeWo (**E**) sub-cell lines in presence of TG2 inhibitors. Survival fractions were normalized to inhibitor-free irradiation-equal controls of each cell line (control). (**F**,**G**) Clonal expansion (CE) after irradiation of A375 (**F**) and MeWo (**G**) sub-cell lines in presence of TG2 inhibitors. Survival fractions were normalized to inhibitor-free irradiation-equal controls of each cell line (control). (**H**) Expansion of spheroidal area of A375-WT and -KO TG2 cells at day 14 after irradiation and treatment with TG2 inhibitors. Spheroidal areas were normalized to inhibitor-free irradiation-equal controls of each cell line (control). Mean values ± SD or mean values alone (when heatmaps were used) from individual experiments (*n* ≥ 4) are shown. Significant variances of treatment-equal samples are displayed as * (TG2 vs. WT, *p* ≤ 0.05), ** (TG2 vs. WT, *p* ≤ 0.01), *** (TG2 vs. WT, *p* ≤ 0.001), ^#^ (TG2 vs. control, *p* ≤ 0.05), and ^##^ (TG2 vs. control, *p* ≤ 0.01).

In order to modify extra- and intracellular TG2 activity, selective irreversible TG2 inhibitors with a *N*^ε^-acryloyl-lysine scaffold were applied. The 6-nitropyridine-2-yl-derivative (**I1**) and the pyridine-4-yl-derivative (**I2**) (structures shown in Figure 4A) were chosen because of diverging membrane permeation rates (P_e_ = 5.1 nm·s^−1^ for **I1** versus P_e_ = 91.6 nm·s^−1^ for **I2**) among comparable inhibitory potential (k_inact_/K_I_ = 10,200 M^−1^·s^−1^ for **I1** versus 10,500 M^−1^·s^−1^ for **I2**) [43]. The lower membrane permeation rate of **I2** permits the discrimination between inhibition of intra- and extracellular TG2.

The treatment of melanoma cell lines A375- and MeWo-WT, with maximum 30 µM of TG2 inhibitor **I1** for up to 72 h, revealed neither cytotoxic effects nor impairment of the cell viability (Figure 4B). Since it is known that the solvent DMSO can activate TG2 [48], the clonal expansion of cells cultured with medium supplemented with or without 35.2 mM DMSO was compared and resulted in no difference (data not shown).

The TG2 inhibitor treatment of A375 sub-cell lines resulted in a reduction in clonal expansion, as compared to inhibitor-free controls, independent of irradiation. This effect was more pronounced for **I1** than for **I2** (Figure 4F). A similar impact of inhibitor treatment was observed in proliferation assays (72 h, Figure 4D). The comparison between the A375 sub-cell lines revealed a less distinct reduction in clonal expansion for A375-TG2 cells, as compared to A375-WT and -control cells, when treated with **I2** and irradiated with 2 Gy.

The clonal expansion of both TG2-naïve and TG2-positive MeWo sub-cell lines was diminished when treated with **I1**, but was unaffected by **I2** treatment (Figure 4G). Furthermore, the comparison between MeWo sub-cell lines revealed a less distinct reduction for MeWo-TG2, as compared to MeWo-WT cells, when irradiated with 2 Gy. Proliferation (72 h) of MeWo-WT and -control cells was reduced after treatment with **I1** and **I2** and subsequent irradiation with 2 Gy, as compared to inhibitor-free controls, in contrast to MeWo-TG2 cells (Figure 4E). This implies that the effects of the inhibitors were independent of the TG2 biosynthesis.

In A375-KO TG2 cells, treatment with **I1** and **I2** diminished the spheroidal expansion equally. Further, this effect was equally distinct with and without irradiation and similar to the effects observed for A375-WT cells (Figure 4H).

## 4. Discussion

In this study, the proliferation in 2D and 3D cultures, as well as the clonal expansion of the melanoma cell lines A375 and MeWo and their descendants, differing in terms of TG2 biosynthesis, were examined in dependency on X-ray irradiation and treatment with TG2 inhibitors. In this context, the clonal expansion assay allows for conclusions about the cell division ability of single cells after treatment, whereas time-dependent effects of the treatment can be detected in proliferation assays. Irradiation dose-dependent effects were clearly distinguishable, providing evidence that the melanoma cell lines are radiosensitive within the applied dose range of between 0 and 4 Gy, and that the applied methods are reliable for the present research approach.

A cellular reaction towards irradiation, proliferation and TG2 biosynthesis was elevated in TG2-overexpressing A375 cells, at different points of time compared to WT cells. Even though enhanced proliferation was also detected in MeWo cells with artificial TG2 biosynthesis, irradiation-dependent changes remained transient and indistinguishable from transduction control cells. In consideration of the irradiation-dependent colony-forming capacity, it can be concluded that an elevated TG2 biosynthesis does not enhance the radioresistance of melanoma cells. Nevertheless, enhancement of TG2 expression (on mRNA level), as a response to irradiation, was also seen in the thymus glands of TG2-deficient mice [49] and murine astrocytes [36].

An opposite strategy was pursued, when TG2 was knocked out by CRISPR/Cas9 technology. In an irradiation-dependent manner, proliferation as well as the clonal and spheroidal expansion of TG2 KO cells were reduced, which provides evidence for a minor contribution of this enzyme to the radioresistance of melanoma cells. This result is in accordance with the observed moderate effects of differential TG2 expression in TG2-positive and -negative melanoma cell lines, on their chemotherapeutic response towards cisplatin and dacarbazine, as reported by Fok et al. [29]. It should be noted that a direct comparison to the mentioned study is not possible because different methods for cell viability assessment were used and the cellular responses to ionizing radiation and DNA-targeting cytostatic agents potentially involve different molecular pathways, even though they are closely related [50]. In lung cancer cells, TG2 was found to be involved in DNA damage repair [16]. This function may also be the underlying mechanism for the results of the present study. In this regard, it was suggested, that other members of the TG family may compensate some of TG2’s functions, especially those linked to the Ca^2+^-dependent TAA, when TG2 is absent [51,52]. Since TG1, TG3 and TG6 biosynthesis was detectable in TG2 KO cells, a partial compensatory effect by these TG family members can be suggested. This might have inversely contributed to the cellular reaction of the TG2 KO cells towards irradiation and thereby, attenuated the effects that were detected here. TG1 and TG3 are known to be involved in the skin barrier function and a synergic activation of TG1 and TG3 together with TG2 was found in UV-irradiated keratinocytes [35,53,54]. This is further supported by our findings associated with the application of TG2 inhibitors. Even though **I1** did not cause cellular toxicity in melanoma cell lines, it reduced the clonal expansion of non-irradiated TG2-naïve MeWo-sub cell lines and the spheroidal expansion of A375-KO TG2 cells. Despite the excellent TG2 selectivity of **I1**, the compound is still reactive towards other TGs [43]. This means, when a sufficient incubation period is given, other TGs might be inhibited by **I1** due to the irreversible binding mode. Consequently, this could explain the observed reduction regarding clonal and spheroidal expansion, independent of the presence of TG2.

In comparison to **I1**, the TG2 inhibitor **I2**, which predominantly targets extracellular TG2 due to a lower membrane permeation rate as compared to **I1**, was employed. A selective inhibition of TG2 was observed by treatment with **I2**, when cellular effects were detected in A375 sub-cell lines, secreting TG2, but not in MeWo sub-cell lines, which do not secrete TG2.

In our study, we found that an increased basal TG2 biosynthesis did not result in higher cellular in vitro TG2 activity. However, in vitro activation of TG2 by irradiation was not tested. Interestingly, other studies evidenced that irradiation-dependent changes in TG2 activity are rather more detectable in vivo/situ than in vitro, and that this effect does not necessarily coincide with changes in TG2 biosynthesis [35,49,55].

## 5. Conclusions

In summary, our results indicate that an increased TG2 biosynthesis does not enhance the radioresistance of melanoma cells, whereas a trend towards impaired proliferation, clonal and spheroidal expansion of TG2 knock out cells, as a response to irradiation, suggests a potential minor contribution of this enzyme to the radioresistance of melanoma cells. Since the biosynthesis of other TG family members in melanoma cells was detected, a partial compensatory effect by these enzymes cannot be excluded. In order to further investigate the involvement of TG2 in the molecular mechanisms of the formation of irradiation resistance in melanoma cells and a potential contribution of TGs, further experiments with single or combined TG KO models, or the use of inhibitors selective for other TGs, should be conducted. Considering the enormous heterogeneity of malignant melanoma at the cell, cell line, and tumor levels, our observation is a basic finding in commonly used specific melanoma cell lines, rather than a generalizable result with immediate specific clinical relevance in melanoma.

## Figures and Tables

**Table 1:** Cell numbers used for set-up of proliferation and clonal expansion assays.

Assay Type	Proliferation	Clonal Expansion			
Irradiation Dose	0–4 Gy	0	0.5	2	4
A375	100,000	500	500	1000	1000
MeWo	200,000	1000	1000	3000	3000

**Table 2:** Antibodies and their specifications used for Western blot analysis.

	Primary Antibodies					Secondary Antibodies	
Target	TG1	TG3	TG6	TG2	ß-Actin	IgG, Rabbit	IgG, Mouse
Source	polyclonal rabbit			monoclonal mouse		polyclonal goat	polyclonal rabbit
Company	Abcam (Cambridge, UK)				Sigma-Aldrich (Steinheim, Germany)		
Product no.	ab103814	ab203229	ab180959	ab2386	A5316	A0545	A9044
Dilution	1:100	1:500	1:1000	1:500	1:1000	1:2000	1:2000/10,000

## Data Availability

The data presented in this study are contained within the article.

## Abbreviations

AKT	Protein kinase B
ATP	Adenosine triphosphate
DNA	Desoxyribonucleic acid
CE	Clonal expansion
DMC	*N*,*N*-dimethylated casein
ECM	Extracellular matrix
FAK	Focal adhesion kinase
GDP	Guanosine diphosphate
GTP	Guanosine triphosphate
I1	*N*^α^-Phenylacetyl-*N*^ε^-acryloyl-l-lysine-4-(6-nitropyridin-2-yl) piperazide
I2	*N*^α^-Phenylacetyl-*N*^ε^-acryloyl-l-lysine-4-(pyridin-4-yl) piperazide
I3	*N*^α^-Phenylacetyl-*N*^ε^-acryloyl-l-lysine-4-(6-methylpyridin-2-yl) piperazide
IL	Interleukin
KO	Knock-out
mRNA	Messenger ribonucleic acid
MT	Mesenchymal transdifferentiation
NF	Nuclear factor
PDGF	Platelet-derived growth factor
PL	Proliferation
SA	Spheroidal area
SD	Standard deviation
SHP	Src homology 2 domain-containing tyrosine Phosphatase
SP	Specificity protein
TAA	Transamidase activity
TG	Transglutaminase
TGF	Transforming growth factor
TNF	Tumor necrosis factor
